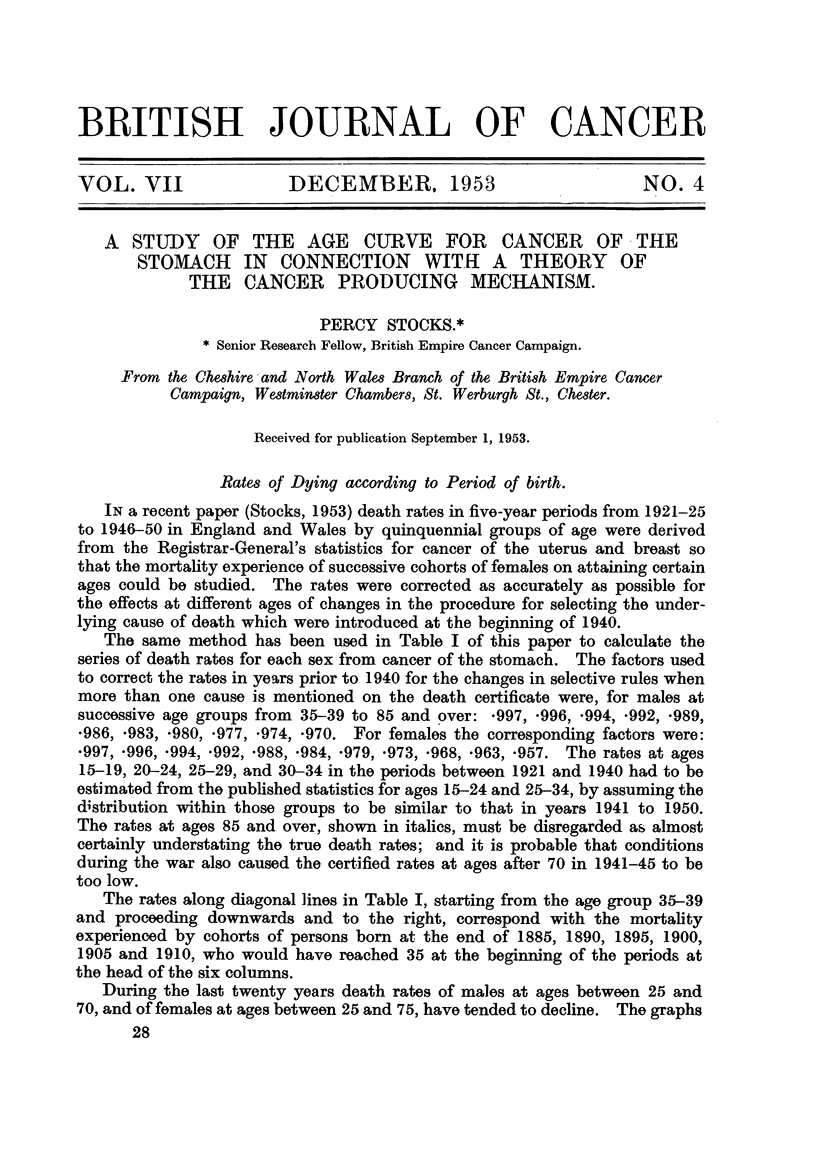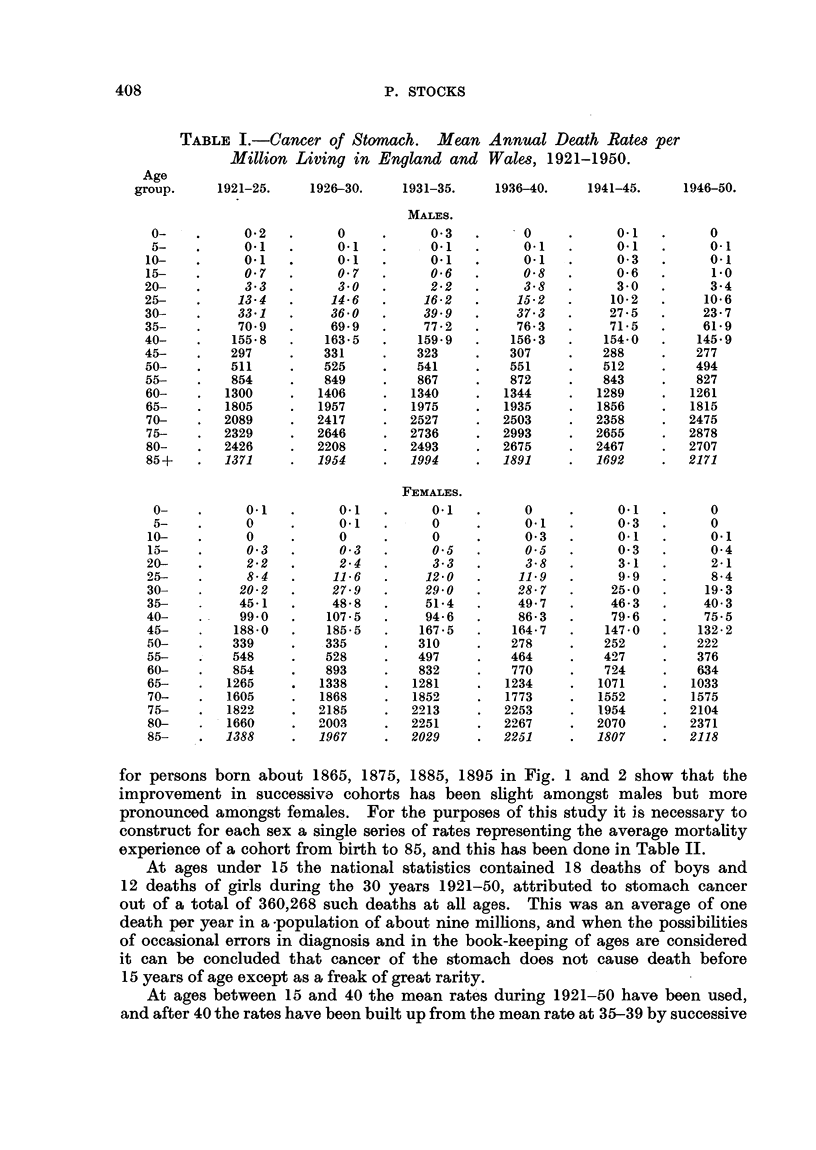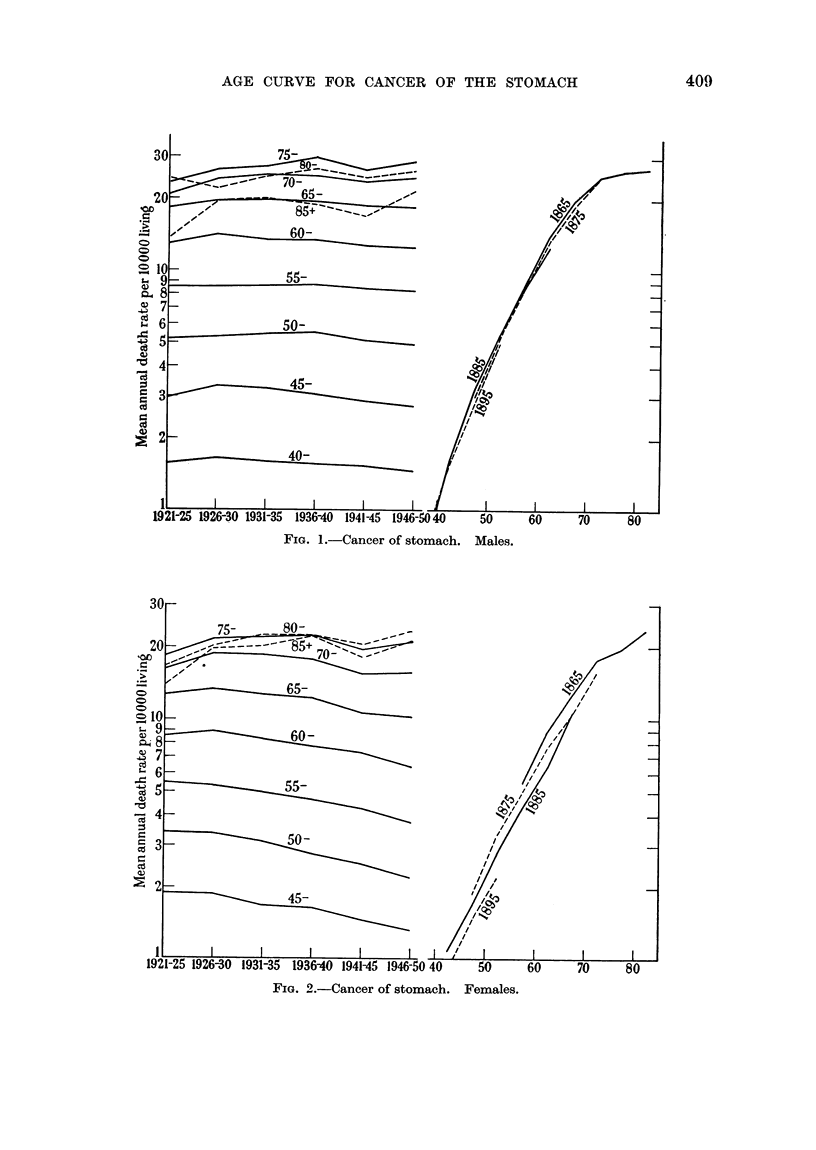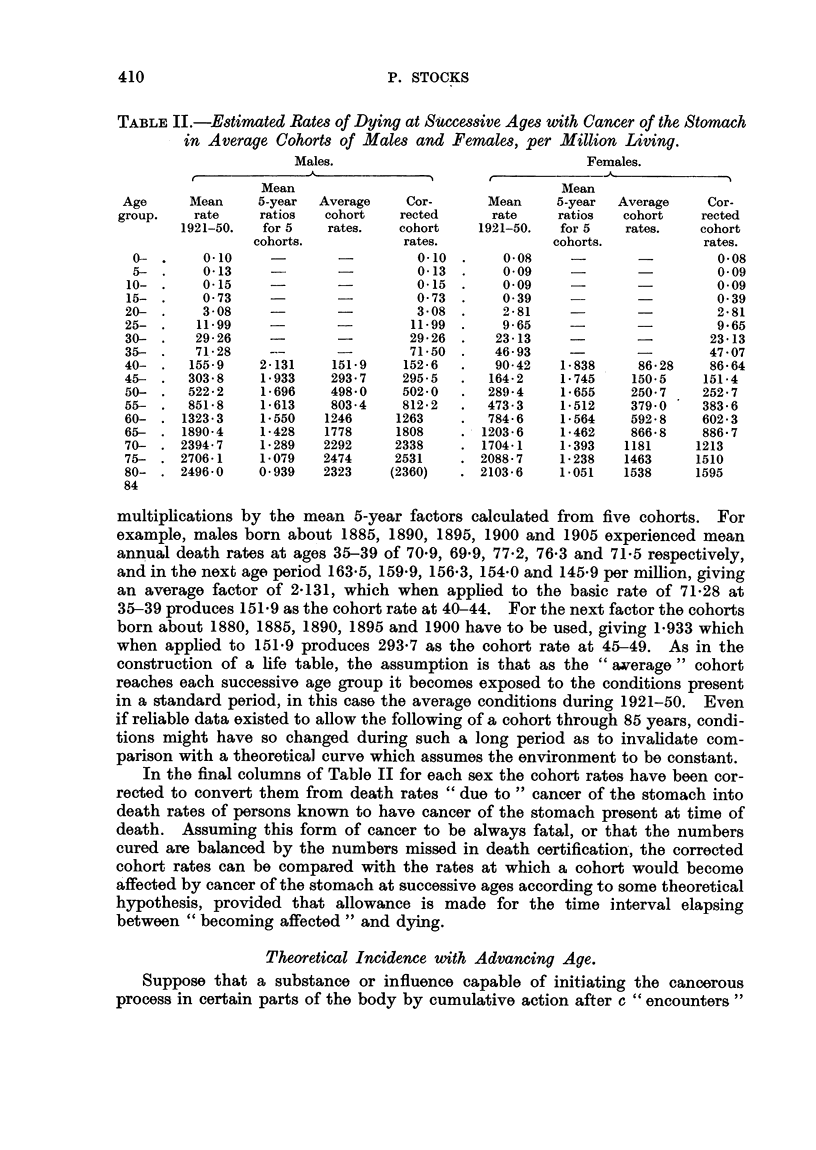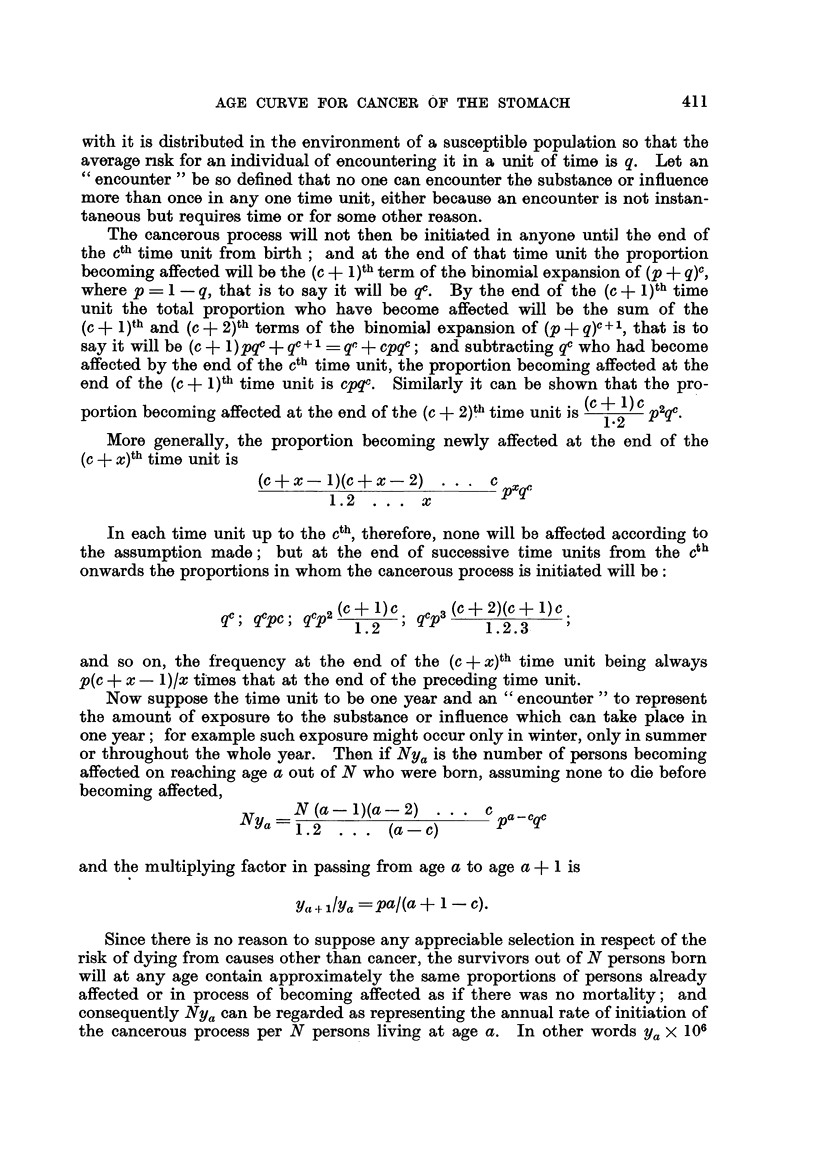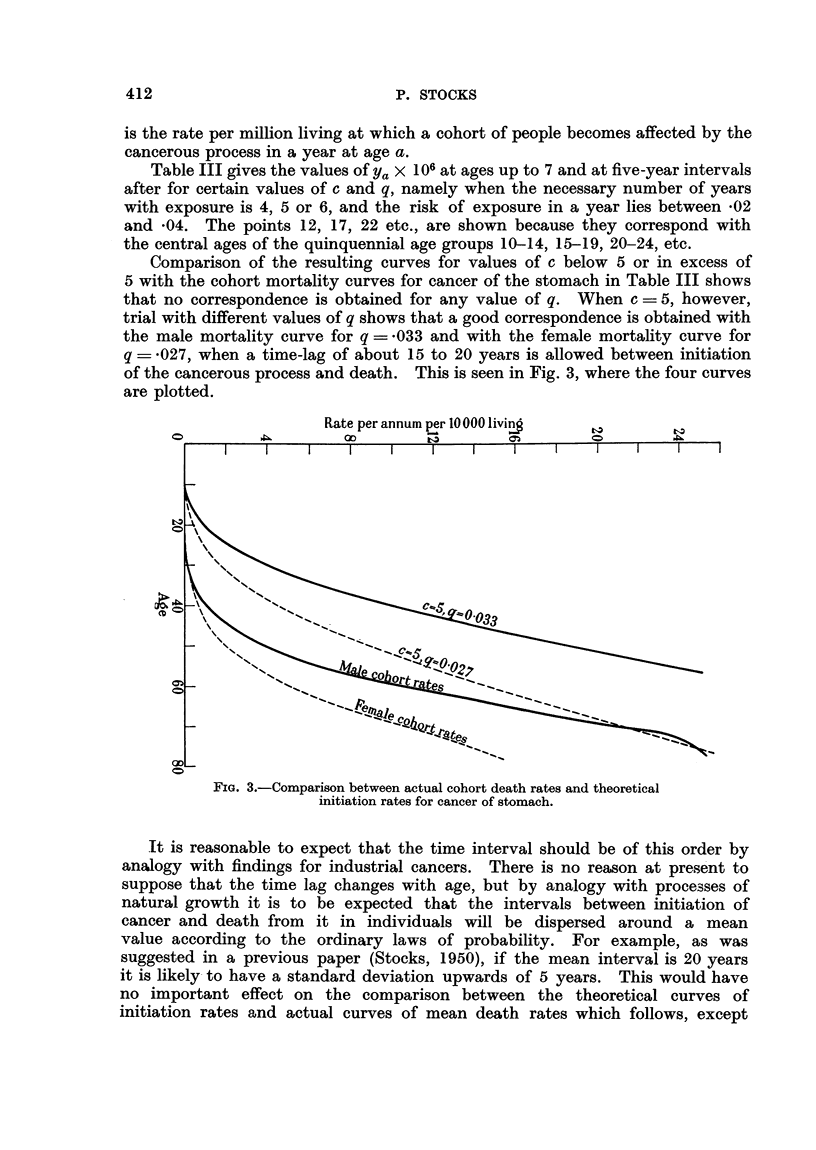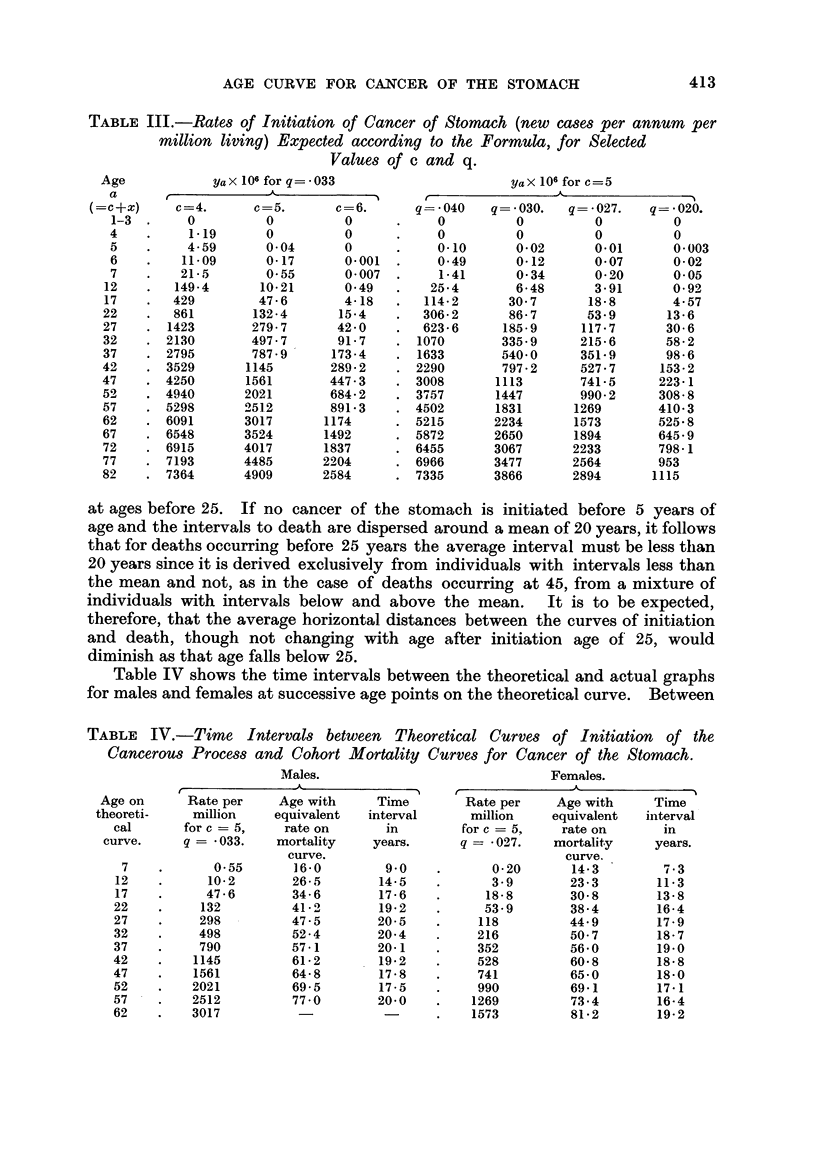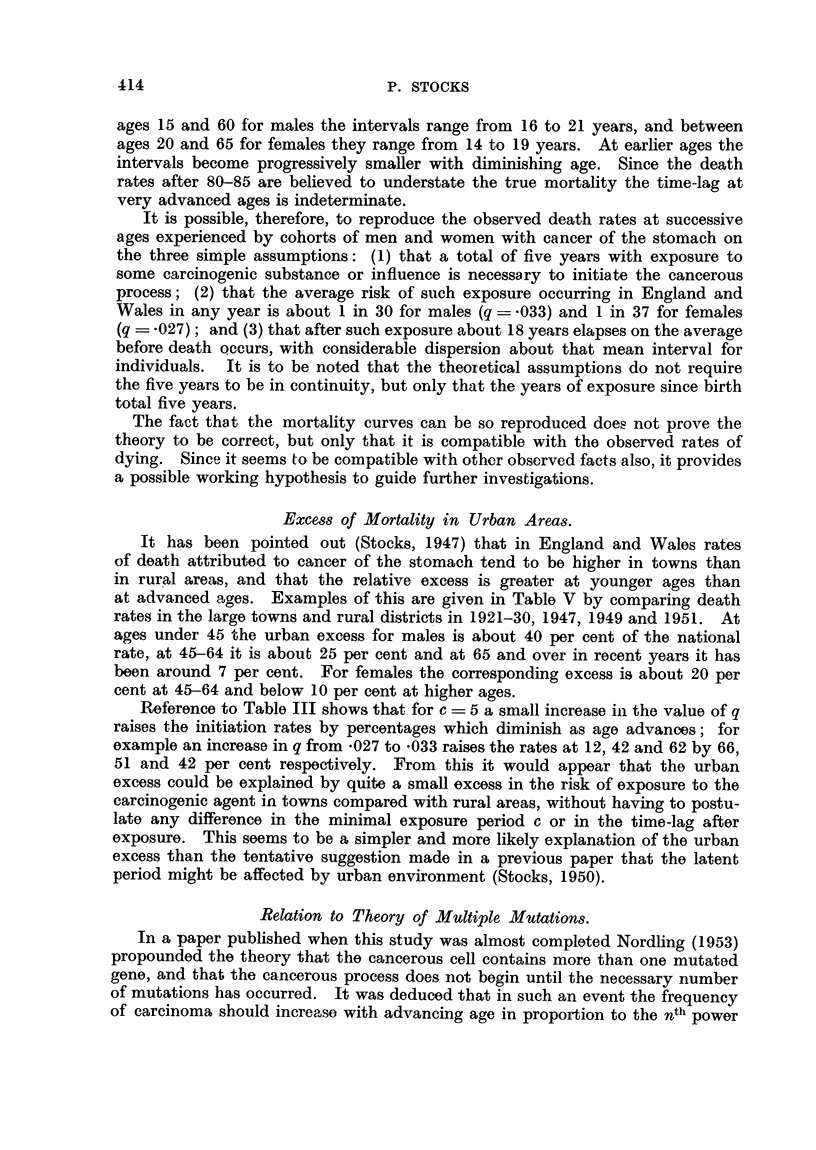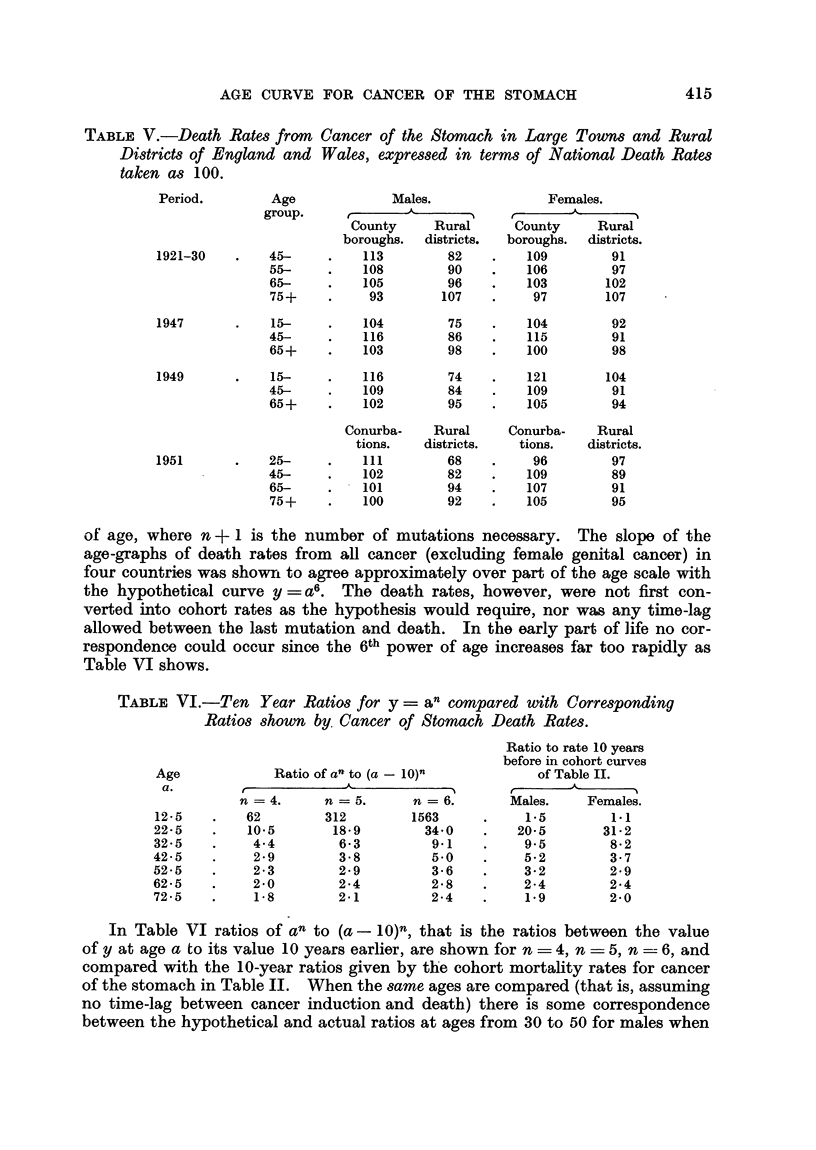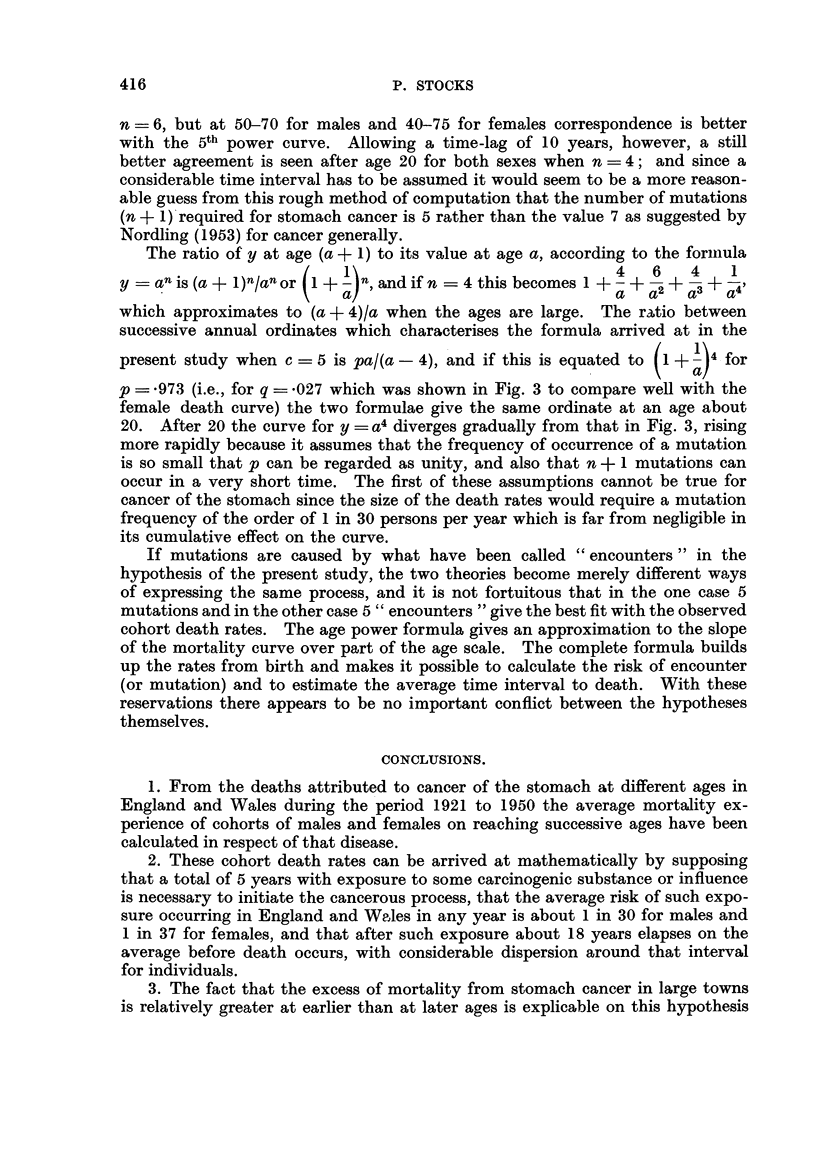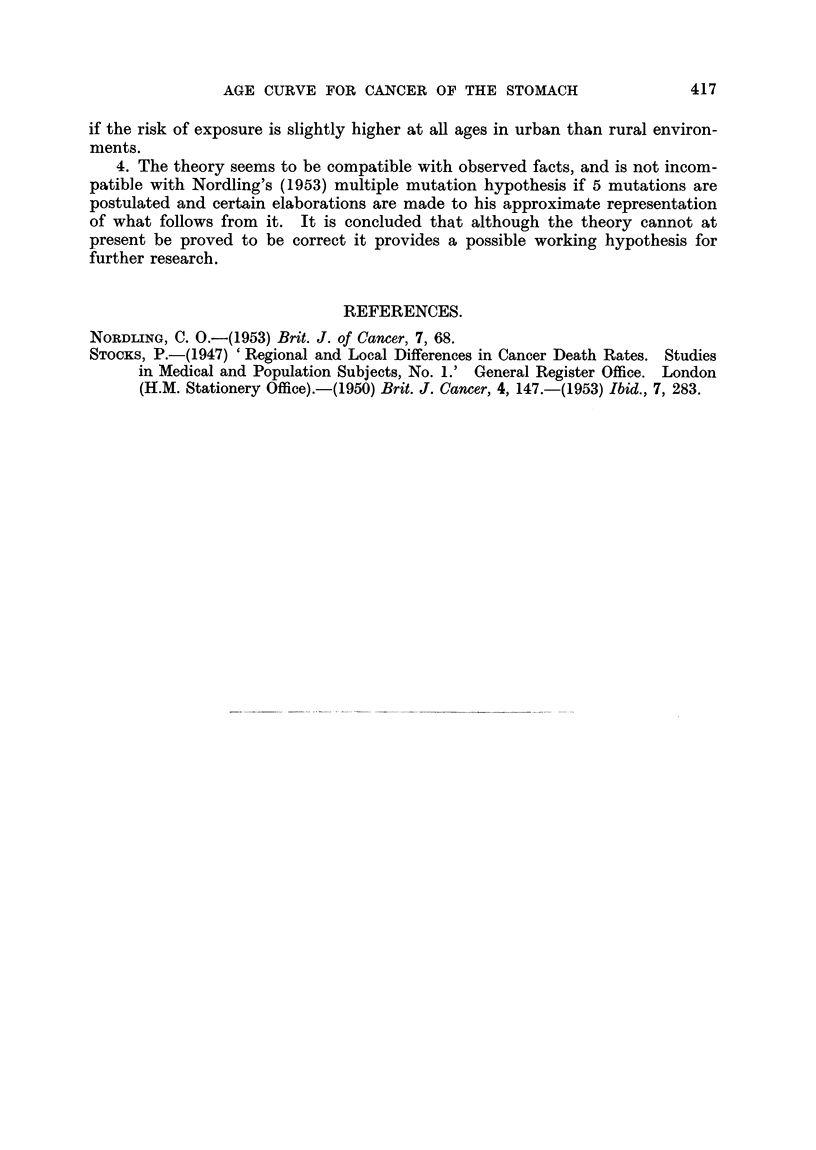# A Study of the Age Curve for Cancer of the Stomach in Connection with a Theory of the Cancer Producing Mechanism

**DOI:** 10.1038/bjc.1953.39

**Published:** 1953-12

**Authors:** Percy Stocks


// BRITISH JOURNAL OF CANCER

VOL. VII DECEMBER, 1953 NO. 4

### A STUDY OF THE AGE CURVE FOR CANCER OF THE STOMACH IN CONNECTION WITH A THEORY OF THE CANCER PRODUCING MECHANISM.

PERCY STOCKS.*

* Senior Research Fellow, British Empire Cancer Campaign.

*From the Cheshire and North Wales Branch of the British Empire Cancer Campaign, Westminster Chambers, St. Werburgh St., Chester.*

Received for publication September 1, 1953.

### *Rates of Dying according to Period of birth.*

IN a recent paper (Stocks, 1953) death rates in five-year periods from 1921–25 to 1946–50 in England and Wales by quinquennial groups of age were derived from the Registrar-General's statistics for cancer of the uterus and breast so that the mortality experience of successive cohorts of females on attaining certain ages could be studied. The rates were corrected as accurately as possible for the effects at different ages of changes in the procedure for selecting the underlying cause of death which were introduced at the beginning of 1940.

The same method has been used in Table I of this paper to calculate the series of death rates for each sex from cancer of the stomach. The factors used to correct the rates in years prior to 1940 for the changes in selective rules when more than one cause is mentioned on the death certificate were, for males at successive age groups from 35–39 to 85 and over: ·997, ·996, ·994, ·992, ·989, ·986, ·983, ·980, ·977, ·974, ·970. For females the corresponding factors were: ·997, ·996, ·994, ·992, ·988, ·984, ·979, ·973, ·968, ·963, ·957. The rates at ages 15–19, 20–24, 25–29, and 30–34 in the periods between 1921 and 1940 had to be estimated from the published statistics for ages 15–24 and 25–34, by assuming the distribution within those groups to be similar to that in years 1941 to 1950. The rates at ages 85 and over, shown in italics, must be disregarded as almost certainly understating the true death rates; and it is probable that conditions during the war also caused the certified rates at ages after 70 in 1941–45 to be too low.

The rates along diagonal lines in Table I, starting from the age group 35–39 and proceeding downwards and to the right, correspond with the mortality experienced by cohorts of persons born at the end of 1885, 1890, 1895, 1900, 1905 and 1910, who would have reached 35 at the beginning of the periods at the head of the six columns.

During the last twenty years death rates of males at ages between 25 and 70, and of females at ages between 25 and 75, have tended to decline. The graphs

TABLE I.—*Cancer of Stomach. Mean Annual Death Rates per Million Living in England and Wales*, 1921–1950.

| Age group. | 1921–25. | 1926–30. | 1931–35. | 1936–40. | 1941–45. | 1946–50. |
|---|---|---|---|---|---|---|
| | | | MALES. | | | |
| 0– | 0·2 | 0 | 0·3 | 0 | 0·1 | 0 |
| 5– | 0·1 | 0·1 | 0·1 | 0·1 | 0·1 | 0·1 |
| 10– | 0·1 | 0·1 | 0·1 | 0·1 | 0·3 | 0·1 |
| 15– | *0·7* | *0·7* | *0·6* | *0·8* | 0·6 | 1·0 |
| 20– | *3·3* | *3·0* | *2·2* | *3·8* | 3·0 | 3·4 |
| 25– | *13·4* | *14·6* | *16·2* | *15·2* | 10·2 | 10·6 |
| 30– | *33·1* | *36·0* | *39·9* | *37·3* | 27·5 | 23·7 |
| 35– | 70·9 | 69·9 | 77·2 | 76·3 | 71·5 | 61·9 |
| 40– | 155·8 | 163·5 | 159·9 | 156·3 | 154·0 | 145·9 |
| 45– | 297 | 331 | 323 | 307 | 288 | 277 |
| 50– | 511 | 525 | 541 | 551 | 512 | 494 |
| 55– | 854 | 849 | 867 | 872 | 843 | 827 |
| 60– | 1300 | 1406 | 1340 | 1344 | 1289 | 1261 |
| 65– | 1805 | 1957 | 1975 | 1935 | 1856 | 1815 |
| 70– | 2089 | 2417 | 2527 | 2503 | 2358 | 2475 |
| 75– | 2329 | 2646 | 2736 | 2993 | 2655 | 2878 |
| 80– | 2426 | 2208 | 2493 | 2675 | 2467 | 2707 |
| 85+ | *1371* | *1954* | *1994* | *1891* | *1692* | *2171* |
| | | | FEMALES. | | | |
| 0– | 0·1 | 0·1 | 0·1 | 0 | 0·1 | 0 |
| 5– | 0 | 0·1 | 0 | 0·1 | 0·3 | 0 |
| 10– | 0 | 0 | 0 | 0·3 | 0·1 | 0·1 |
| 15– | *0·3* | *0·3* | *0·5* | *0·5* | 0·3 | 0·4 |
| 20– | *2·2* | *2·4* | *3·3* | *3·8* | 3·1 | 2·1 |
| 25– | *8·4* | *11·6* | *12·0* | *11·9* | 9·9 | 8·4 |
| 30– | *20·2* | *27·9* | *29·0* | *28·7* | 25·0 | 19·3 |
| 35– | 45·1 | 48·8 | 51·4 | 49·7 | 46·3 | 40·3 |
| 40– | 99·0 | 107·5 | 94·6 | 86·3 | 79·6 | 75·5 |
| 45– | 188·0 | 185·5 | 167·5 | 164·7 | 147·0 | 132·2 |
| 50– | 339 | 335 | 310 | 278 | 252 | 222 |
| 55– | 548 | 528 | 497 | 464 | 427 | 376 |
| 60– | 854 | 893 | 832 | 770 | 724 | 634 |
| 65– | 1265 | 1338 | 1281 | 1234 | 1071 | 1033 |
| 70– | 1605 | 1868 | 1852 | 1773 | 1552 | 1575 |
| 75– | 1822 | 2185 | 2213 | 2253 | 1954 | 2104 |
| 80– | 1660 | 2003 | 2251 | 2267 | 2070 | 2371 |
| 85– | *1388* | *1967* | *2029* | *2251* | *1807* | *2118* |

for persons born about 1865, 1875, 1885, 1895 in Fig. 1 and 2 show that the improvement in successive cohorts has been slight amongst males but more pronounced amongst females. For the purposes of this study it is necessary to construct for each sex a single series of rates representing the average mortality experience of a cohort from birth to 85, and this has been done in Table II.

At ages under 15 the national statistics contained 18 deaths of boys and 12 deaths of girls during the 30 years 1921–50, attributed to stomach cancer out of a total of 360,268 such deaths at all ages. This was an average of one death per year in a population of about nine millions, and when the possibilities of occasional errors in diagnosis and in the book-keeping of ages are considered it can be concluded that cancer of the stomach does not cause death before 15 years of age except as a freak of great rarity.

At ages between 15 and 40 the mean rates during 1921–50 have been used, and after 40 the rates have been built up from the mean rate at 35–39 by successive

FIG. 1.—Cancer of stomach. Males.

FIG. 2.—Cancer of stomach. Females.

TABLE II.—*Estimated Rates of Dying at Successive Ages with Cancer of the Stomach in Average Cohorts of Males and Females, per Million Living.*

| Age group. | Males. Mean rate 1921–50. | Males. Mean 5-year ratios for 5 cohorts. | Males. Average cohort rates. | Males. Corrected cohort rates. | Females. Mean rate 1921–50. | Females. Mean 5-year ratios for 5 cohorts. | Females. Average cohort rates. | Females. Corrected cohort rates. |
|---|---|---|---|---|---|---|---|---|
| 0– | 0·10 | — | — | 0·10 | 0·08 | — | — | 0·08 |
| 5– | 0·13 | — | — | 0·13 | 0·09 | — | — | 0·09 |
| 10– | 0·15 | — | — | 0·15 | 0·09 | — | — | 0·09 |
| 15– | 0·73 | — | — | 0·73 | 0·39 | — | — | 0·39 |
| 20– | 3·08 | — | — | 3·08 | 2·81 | — | — | 2·81 |
| 25– | 11·99 | — | — | 11·99 | 9·65 | — | — | 9·65 |
| 30– | 29·26 | — | — | 29·26 | 23·13 | — | — | 23·13 |
| 35– | 71·28 | — | — | 71·50 | 46·93 | — | — | 47·07 |
| 40– | 155·9 | 2·131 | 151·9 | 152·6 | 90·42 | 1·838 | 86·28 | 86·64 |
| 45– | 303·8 | 1·933 | 293·7 | 295·5 | 164·2 | 1·745 | 150·5 | 151·4 |
| 50– | 522·2 | 1·696 | 498·0 | 502·0 | 289·4 | 1·655 | 250·7 | 252·7 |
| 55– | 851·8 | 1·613 | 803·4 | 812·2 | 473·3 | 1·512 | 379·0 | 383·6 |
| 60– | 1323·3 | 1·550 | 1246 | 1263 | 784·6 | 1·564 | 592·8 | 602·3 |
| 65– | 1890·4 | 1·428 | 1778 | 1808 | 1203·6 | 1·462 | 866·8 | 886·7 |
| 70– | 2394·7 | 1·289 | 2292 | 2338 | 1704·1 | 1·393 | 1181 | 1213 |
| 75– | 2706·1 | 1·079 | 2474 | 2531 | 2088·7 | 1·238 | 1463 | 1510 |
| 80–84 | 2496·0 | 0·939 | 2323 | (2360) | 2103·6 | 1·051 | 1538 | 1595 |

multiplications by the mean 5-year factors calculated from five cohorts. For example, males born about 1885, 1890, 1895, 1900 and 1905 experienced mean annual death rates at ages 35–39 of 70·9, 69·9, 77·2, 76·3 and 71·5 respectively, and in the next age period 163·5, 159·9, 156·3, 154·0 and 145·9 per million, giving an average factor of 2·131, which when applied to the basic rate of 71·28 at 35–39 produces 151·9 as the cohort rate at 40–44. For the next factor the cohorts born about 1880, 1885, 1890, 1895 and 1900 have to be used, giving 1·933 which when applied to 151·9 produces 293·7 as the cohort rate at 45–49. As in the construction of a life table, the assumption is that as the " average " cohort reaches each successive age group it becomes exposed to the conditions present in a standard period, in this case the average conditions during 1921–50. Even if reliable data existed to allow the following of a cohort through 85 years, conditions might have so changed during such a long period as to invalidate comparison with a theoretical curve which assumes the environment to be constant.

In the final columns of Table II for each sex the cohort rates have been corrected to convert them from death rates " due to " cancer of the stomach into death rates of persons known to have cancer of the stomach present at time of death. Assuming this form of cancer to be always fatal, or that the numbers cured are balanced by the numbers missed in death certification, the corrected cohort rates can be compared with the rates at which a cohort would become affected by cancer of the stomach at successive ages according to some theoretical hypothesis, provided that allowance is made for the time interval elapsing between " becoming affected " and dying.

### *Theoretical Incidence with Advancing Age.*

Suppose that a substance or influence capable of initiating the cancerous process in certain parts of the body by cumulative action after $c$ " encounters "

with it is distributed in the environment of a susceptible population so that the average risk for an individual of encountering it in a unit of time is $q$. Let an "encounter" be so defined that no one can encounter the substance or influence more than once in any one time unit, either because an encounter is not instantaneous but requires time or for some other reason.

The cancerous process will not then be initiated in anyone until the end of the $c^{th}$ time unit from birth ; and at the end of that time unit the proportion becoming affected will be the $(c+1)^{th}$ term of the binomial expansion of $(p+q)^c$, where $p=1-q$, that is to say it will be $q^c$. By the end of the $(c+1)^{th}$ time unit the total proportion who have become affected will be the sum of the $(c+1)^{th}$ and $(c+2)^{th}$ terms of the binomial expansion of $(p+q)^{c+1}$, that is to say it will be $(c+1)pq^c+q^{c+1}=q^c+cpq^c$ ; and subtracting $q^c$ who had become affected by the end of the $c^{th}$ time unit, the proportion becoming affected at the end of the $(c+1)^{th}$ time unit is $cpq^c$. Similarly it can be shown that the proportion becoming affected at the end of the $(c+2)^{th}$ time unit is $\frac{(c+1)c}{1\cdot 2}p^2q^c$.

More generally, the proportion becoming newly affected at the end of the $(c+x)^{th}$ time unit is

$$\frac{(c+x-1)(c+x-2)\ \ldots\ c}{1.2\ \ldots\ x}p^xq^c$$

In each time unit up to the $c^{th}$, therefore, none will be affected according to the assumption made; but at the end of successive time units from the $c^{th}$ onwards the proportions in whom the cancerous process is initiated will be:

$$q^c;\ q^cpc;\ q^cp^2\frac{(c+1)c}{1.2};\ q^cp^3\frac{(c+2)(c+1)c}{1.2.3};$$

and so on, the frequency at the end of the $(c+x)^{th}$ time unit being always $p(c+x-1)/x$ times that at the end of the preceding time unit.

Now suppose the time unit to be one year and an "encounter" to represent the amount of exposure to the substance or influence which can take place in one year ; for example such exposure might occur only in winter, only in summer or throughout the whole year. Then if $Ny_a$ is the number of persons becoming affected on reaching age $a$ out of $N$ who were born, assuming none to die before becoming affected,

$$Ny_a=\frac{N\ (a-1)(a-2)\ \ldots\ c}{1.2\ \ldots\ (a-c)}p^{a-c}q^c$$

and the multiplying factor in passing from age $a$ to age $a+1$ is

$$y_{a+1}/y_a=pa/(a+1-c).$$

Since there is no reason to suppose any appreciable selection in respect of the risk of dying from causes other than cancer, the survivors out of $N$ persons born will at any age contain approximately the same proportions of persons already affected or in process of becoming affected as if there was no mortality; and consequently $Ny_a$ can be regarded as representing the annual rate of initiation of the cancerous process per $N$ persons living at age $a$. In other words $y_a\times 10^6$

is the rate per million living at which a cohort of people becomes affected by the cancerous process in a year at age $a$.

Table III gives the values of $y_a \times 10^6$ at ages up to 7 and at five-year intervals after for certain values of $c$ and $q$, namely when the necessary number of years with exposure is 4, 5 or 6, and the risk of exposure in a year lies between ·02 and ·04. The points 12, 17, 22 etc., are shown because they correspond with the central ages of the quinquennial age groups 10–14, 15–19, 20–24, etc.

Comparison of the resulting curves for values of $c$ below 5 or in excess of 5 with the cohort mortality curves for cancer of the stomach in Table III shows that no correspondence is obtained for any value of $q$. When $c = 5$, however, trial with different values of $q$ shows that a good correspondence is obtained with the male mortality curve for $q = ·033$ and with the female mortality curve for $q = ·027$, when a time-lag of about 15 to 20 years is allowed between initiation of the cancerous process and death. This is seen in Fig. 3, where the four curves are plotted.

Fig. 3.—Comparison between actual cohort death rates and theoretical initiation rates for cancer of stomach.

It is reasonable to expect that the time interval should be of this order by analogy with findings for industrial cancers. There is no reason at present to suppose that the time lag changes with age, but by analogy with processes of natural growth it is to be expected that the intervals between initiation of cancer and death from it in individuals will be dispersed around a mean value according to the ordinary laws of probability. For example, as was suggested in a previous paper (Stocks, 1950), if the mean interval is 20 years it is likely to have a standard deviation upwards of 5 years. This would have no important effect on the comparison between the theoretical curves of initiation rates and actual curves of mean death rates which follows, except

TABLE III.—*Rates of Initiation of Cancer of Stomach (new cases per annum per million living) Expected according to the Formula, for Selected Values of* c *and* q.

| Age $a$ (=$c+x$) | $y_a\times 10^6$ for $q=\cdot 033$: $c=4.$ | $c=5.$ | $c=6.$ | $y_a\times 10^6$ for $c=5$: $q=\cdot 040$ | $q=\cdot 030.$ | $q=\cdot 027.$ | $q=\cdot 020.$ |
|---|---|---|---|---|---|---|---|
| 1–3 | 0 | 0 | 0 | 0 | 0 | 0 | 0 |
| 4 | 1·19 | 0 | 0 | 0 | 0 | 0 | 0 |
| 5 | 4·59 | 0·04 | 0 | 0·10 | 0·02 | 0·01 | 0·003 |
| 6 | 11·09 | 0·17 | 0·001 | 0·49 | 0·12 | 0·07 | 0·02 |
| 7 | 21·5 | 0·55 | 0·007 | 1·41 | 0·34 | 0·20 | 0·05 |
| 12 | 149·4 | 10·21 | 0·49 | 25·4 | 6·48 | 3·91 | 0·92 |
| 17 | 429 | 47·6 | 4·18 | 114·2 | 30·7 | 18·8 | 4·57 |
| 22 | 861 | 132·4 | 15·4 | 306·2 | 86·7 | 53·9 | 13·6 |
| 27 | 1423 | 279·7 | 42·0 | 623·6 | 185·9 | 117·7 | 30·6 |
| 32 | 2130 | 497·7 | 91·7 | 1070 | 335·9 | 215·6 | 58·2 |
| 37 | 2795 | 787·9 | 173·4 | 1633 | 540·0 | 351·9 | 98·6 |
| 42 | 3529 | 1145 | 289·2 | 2290 | 797·2 | 527·7 | 153·2 |
| 47 | 4250 | 1561 | 447·3 | 3008 | 1113 | 741·5 | 223·1 |
| 52 | 4940 | 2021 | 684·2 | 3757 | 1447 | 990·2 | 308·8 |
| 57 | 5298 | 2512 | 891·3 | 4502 | 1831 | 1269 | 410·3 |
| 62 | 6091 | 3017 | 1174 | 5215 | 2234 | 1573 | 525·8 |
| 67 | 6548 | 3524 | 1492 | 5872 | 2650 | 1894 | 645·9 |
| 72 | 6915 | 4017 | 1837 | 6455 | 3067 | 2233 | 798·1 |
| 77 | 7193 | 4485 | 2204 | 6966 | 3477 | 2564 | 953 |
| 82 | 7364 | 4909 | 2584 | 7335 | 3866 | 2894 | 1115 |

at ages before 25. If no cancer of the stomach is initiated before 5 years of age and the intervals to death are dispersed around a mean of 20 years, it follows that for deaths occurring before 25 years the average interval must be less than 20 years since it is derived exclusively from individuals with intervals less than the mean and not, as in the case of deaths occurring at 45, from a mixture of individuals with intervals below and above the mean. It is to be expected, therefore, that the average horizontal distances between the curves of initiation and death, though not changing with age after initiation age of 25, would diminish as that age falls below 25.

Table IV shows the time intervals between the theoretical and actual graphs for males and females at successive age points on the theoretical curve. Between

TABLE IV.—*Time Intervals between Theoretical Curves of Initiation of the Cancerous Process and Cohort Mortality Curves for Cancer of the Stomach.*

| Age on theoretical curve. | Males. Rate per million for $c = 5$, $q = \cdot 033$. | Males. Age with equivalent rate on mortality curve. | Males. Time interval in years. | Females. Rate per million for $c = 5$, $q = \cdot 027$. | Females. Age with equivalent rate on mortality curve. | Females. Time interval in years. |
|---|---|---|---|---|---|---|
| 7 | 0·55 | 16·0 | 9·0 | 0·20 | 14·3 | 7·3 |
| 12 | 10·2 | 26·5 | 14·5 | 3·9 | 23·3 | 11·3 |
| 17 | 47·6 | 34·6 | 17·6 | 18·8 | 30·8 | 13·8 |
| 22 | 132 | 41·2 | 19·2 | 53·9 | 38·4 | 16·4 |
| 27 | 298 | 47·5 | 20·5 | 118 | 44·9 | 17·9 |
| 32 | 498 | 52·4 | 20·4 | 216 | 50·7 | 18·7 |
| 37 | 790 | 57·1 | 20·1 | 352 | 56·0 | 19·0 |
| 42 | 1145 | 61·2 | 19·2 | 528 | 60·8 | 18·8 |
| 47 | 1561 | 64·8 | 17·8 | 741 | 65·0 | 18·0 |
| 52 | 2021 | 69·5 | 17·5 | 990 | 69·1 | 17·1 |
| 57 | 2512 | 77·0 | 20·0 | 1269 | 73·4 | 16·4 |
| 62 | 3017 | — | — | 1573 | 81·2 | 19·2 |

ages 15 and 60 for males the intervals range from 16 to 21 years, and between ages 20 and 65 for females they range from 14 to 19 years. At earlier ages the intervals become progressively smaller with diminishing age. Since the death rates after 80–85 are believed to understate the true mortality the time-lag at very advanced ages is indeterminate.

It is possible, therefore, to reproduce the observed death rates at successive ages experienced by cohorts of men and women with cancer of the stomach on the three simple assumptions: (1) that a total of five years with exposure to some carcinogenic substance or influence is necessary to initiate the cancerous process; (2) that the average risk of such exposure occurring in England and Wales in any year is about 1 in 30 for males ($q = \cdot 033$) and 1 in 37 for females ($q = \cdot 027$); and (3) that after such exposure about 18 years elapses on the average before death occurs, with considerable dispersion about that mean interval for individuals. It is to be noted that the theoretical assumptions do not require the five years to be in continuity, but only that the years of exposure since birth total five years.

The fact that the mortality curves can be so reproduced does not prove the theory to be correct, but only that it is compatible with the observed rates of dying. Since it seems to be compatible with other observed facts also, it provides a possible working hypothesis to guide further investigations.

*Excess of Mortality in Urban Areas.*

It has been pointed out (Stocks, 1947) that in England and Wales rates of death attributed to cancer of the stomach tend to be higher in towns than in rural areas, and that the relative excess is greater at younger ages than at advanced ages. Examples of this are given in Table V by comparing death rates in the large towns and rural districts in 1921–30, 1947, 1949 and 1951. At ages under 45 the urban excess for males is about 40 per cent of the national rate, at 45–64 it is about 25 per cent and at 65 and over in recent years it has been around 7 per cent. For females the corresponding excess is about 20 per cent at 45–64 and below 10 per cent at higher ages.

Reference to Table III shows that for $c = 5$ a small increase in the value of $q$ raises the initiation rates by percentages which diminish as age advances; for example an increase in $q$ from ·027 to ·033 raises the rates at 12, 42 and 62 by 66, 51 and 42 per cent respectively. From this it would appear that the urban excess could be explained by quite a small excess in the risk of exposure to the carcinogenic agent in towns compared with rural areas, without having to postulate any difference in the minimal exposure period $c$ or in the time-lag after exposure. This seems to be a simpler and more likely explanation of the urban excess than the tentative suggestion made in a previous paper that the latent period might be affected by urban environment (Stocks, 1950).

*Relation to Theory of Multiple Mutations.*

In a paper published when this study was almost completed Nordling (1953) propounded the theory that the cancerous cell contains more than one mutated gene, and that the cancerous process does not begin until the necessary number of mutations has occurred. It was deduced that in such an event the frequency of carcinoma should increase with advancing age in proportion to the $n^{\text{th}}$ power

TABLE V.—*Death Rates from Cancer of the Stomach in Large Towns and Rural Districts of England and Wales, expressed in terms of National Death Rates taken as* 100.

| Period. | Age group. | Males. | | Females. | |
|---|---|---|---|---|---|
| | | County boroughs. | Rural districts. | County boroughs. | Rural districts. |
| 1921–30 | 45– | 113 | 82 | 109 | 91 |
| | 55– | 108 | 90 | 106 | 97 |
| | 65– | 105 | 96 | 103 | 102 |
| | 75+ | 93 | 107 | 97 | 107 |
| 1947 | 15– | 104 | 75 | 104 | 92 |
| | 45– | 116 | 86 | 115 | 91 |
| | 65+ | 103 | 98 | 100 | 98 |
| 1949 | 15– | 116 | 74 | 121 | 104 |
| | 45– | 109 | 84 | 109 | 91 |
| | 65+ | 102 | 95 | 105 | 94 |
| | | Conurbations. | Rural districts. | Conurbations. | Rural districts. |
| 1951 | 25– | 111 | 68 | 96 | 97 |
| | 45– | 102 | 82 | 109 | 89 |
| | 65– | 101 | 94 | 107 | 91 |
| | 75+ | 100 | 92 | 105 | 95 |

of age, where $n + 1$ is the number of mutations necessary. The slope of the age-graphs of death rates from all cancer (excluding female genital cancer) in four countries was shown to agree approximately over part of the age scale with the hypothetical curve $y = a^6$. The death rates, however, were not first converted into cohort rates as the hypothesis would require, nor was any time-lag allowed between the last mutation and death. In the early part of life no correspondence could occur since the $6^{th}$ power of age increases far too rapidly as Table VI shows.

TABLE VI.—*Ten Year Ratios for* $y = a^n$ *compared with Corresponding Ratios shown by Cancer of Stomach Death Rates.*

| Age $a$. | Ratio of $a^n$ to $(a - 10)^n$ | | | Ratio to rate 10 years before in cohort curves of Table II. | |
|---|---|---|---|---|---|
| | $n = 4$. | $n = 5$. | $n = 6$. | Males. | Females. |
| 12·5 | 62 | 312 | 1563 | 1·5 | 1·1 |
| 22·5 | 10·5 | 18·9 | 34·0 | 20·5 | 31·2 |
| 32·5 | 4·4 | 6·3 | 9·1 | 9·5 | 8·2 |
| 42·5 | 2·9 | 3·8 | 5·0 | 5·2 | 3·7 |
| 52·5 | 2·3 | 2·9 | 3·6 | 3·2 | 2·9 |
| 62·5 | 2·0 | 2·4 | 2·8 | 2·4 | 2·4 |
| 72·5 | 1·8 | 2·1 | 2·4 | 1·9 | 2·0 |

In Table VI ratios of $a^n$ to $(a - 10)^n$, that is the ratios between the value of $y$ at age $a$ to its value 10 years earlier, are shown for $n = 4$, $n = 5$, $n = 6$, and compared with the 10-year ratios given by the cohort mortality rates for cancer of the stomach in Table II. When the *same* ages are compared (that is, assuming no time-lag between cancer induction and death) there is some correspondence between the hypothetical and actual ratios at ages from 30 to 50 for males when

$n = 6$, but at 50–70 for males and 40–75 for females correspondence is better with the 5th power curve. Allowing a time-lag of 10 years, however, a still better agreement is seen after age 20 for both sexes when $n = 4$; and since a considerable time interval has to be assumed it would seem to be a more reasonable guess from this rough method of computation that the number of mutations $(n + 1)$ required for stomach cancer is 5 rather than the value 7 as suggested by Nordling (1953) for cancer generally.

The ratio of $y$ at age $(a + 1)$ to its value at age $a$, according to the formula $y = a^n$ is $(a + 1)^n/a^n$ or $\left(1 + \frac{1}{a}\right)^n$, and if $n = 4$ this becomes $1 + \frac{4}{a} + \frac{6}{a^2} + \frac{4}{a^3} + \frac{1}{a^4}$, which approximates to $(a + 4)/a$ when the ages are large. The ratio between successive annual ordinates which characterises the formula arrived at in the present study when $c = 5$ is $pa/(a - 4)$, and if this is equated to $\left(1 + \frac{1}{a}\right)^4$ for $p = \cdot973$ (i.e., for $q = \cdot027$ which was shown in Fig. 3 to compare well with the female death curve) the two formulae give the same ordinate at an age about 20. After 20 the curve for $y = a^4$ diverges gradually from that in Fig. 3, rising more rapidly because it assumes that the frequency of occurrence of a mutation is so small that $p$ can be regarded as unity, and also that $n + 1$ mutations can occur in a very short time. The first of these assumptions cannot be true for cancer of the stomach since the size of the death rates would require a mutation frequency of the order of 1 in 30 persons per year which is far from negligible in its cumulative effect on the curve.

If mutations are caused by what have been called "encounters" in the hypothesis of the present study, the two theories become merely different ways of expressing the same process, and it is not fortuitous that in the one case 5 mutations and in the other case 5 "encounters" give the best fit with the observed cohort death rates. The age power formula gives an approximation to the slope of the mortality curve over part of the age scale. The complete formula builds up the rates from birth and makes it possible to calculate the risk of encounter (or mutation) and to estimate the average time interval to death. With these reservations there appears to be no important conflict between the hypotheses themselves.

### CONCLUSIONS.

1. From the deaths attributed to cancer of the stomach at different ages in England and Wales during the period 1921 to 1950 the average mortality experience of cohorts of males and females on reaching successive ages have been calculated in respect of that disease.

2. These cohort death rates can be arrived at mathematically by supposing that a total of 5 years with exposure to some carcinogenic substance or influence is necessary to initiate the cancerous process, that the average risk of such exposure occurring in England and Wales in any year is about 1 in 30 for males and 1 in 37 for females, and that after such exposure about 18 years elapses on the average before death occurs, with considerable dispersion around that interval for individuals.

3. The fact that the excess of mortality from stomach cancer in large towns is relatively greater at earlier than at later ages is explicable on this hypothesis if the risk of exposure is slightly higher at all ages in urban than rural environments.

4. The theory seems to be compatible with observed facts, and is not incompatible with Nordling's (1953) multiple mutation hypothesis if 5 mutations are postulated and certain elaborations are made to his approximate representation of what follows from it. It is concluded that although the theory cannot at present be proved to be correct it provides a possible working hypothesis for further research.

### REFERENCES.

NORDLING, C. O.—(1953) *Brit. J. of Cancer*, **7**, 68.
STOCKS, P.—(1947) 'Regional and Local Differences in Cancer Death Rates. Studies in Medical and Population Subjects, No. 1.' General Register Office. London (H.M. Stationery Office).—(1950) *Brit. J. Cancer*, **4**, 147.—(1953) *Ibid.*, **7**, 283.